# Dr. Sivaramakrishna Iyer Padmavati: The Queen of Hearts

**DOI:** 10.7759/cureus.71563

**Published:** 2024-10-15

**Authors:** Sahar Shafique, Hannah G VanHoozier, Amanda Ruiz, Jason Perez, Naunihal T Zaveri, Latha Ganti

**Affiliations:** 1 Research, Orlando College of Osteopathic Medicine, Winter Garden, USA; 2 Biomedical Sciences, Orlando College of Osteopathic Medicine, Winter Garden, USA; 3 Emergency Medicine and Neurology, University of Central Florida, Orlando, USA; 4 Medical Science, The Warren Alpert Medical School of Brown University, Providence, USA

**Keywords:** biographies, cardiology, female cardiologist, historical vignette, indian doctors, innovation in medicine, medical biographies, medical innovation, medical stories, women in medicine

## Abstract

Dr. Sivaramakrishna Iyer Padmavati was the first female cardiologist in India. She set the foundation for cardiovascular treatment in India, going above and beyond to seek extensive cardiovascular education. She overcame gender-based limitations, paving the way for more equitable opportunities for women in India. Her efforts to spread knowledge about cardiology to the government and hospitals simultaneously caused her to advocate healthcare for Indian citizens. She transformed healthcare through hard work, innovative thinking, and unwavering dedication. Her hard work and passion for cardiology transformed healthcare in India.

## Introduction and background

Early life and career

For over a century, Dr. Sivaramakrishna Iyer Padmavati worked hard to pave the way for the future of cardiology and medicine as a whole. Born in Burma (now known as Myanmar), Dr. Padmavati's legacy started from a young age [1]. She was an active child, participating in various sports, most notably swimming. From a young age, she learned about health and wellness. As the first female student at the Rangoon Medical College, Dr. Padmavati earned her Bachelor of Medicine and Bachelor of Surgery (MBBS) degree with prestigious honors. Becoming a female physician at this time was relatively uncommon, demonstrating Dr. Padmavati's bravery and courage to dive head-first into the world of medicine [2,3].

In 1942, during World War II, the hometown of Padmavati and her family was invaded by Japan, forcing her, her mother, and her sisters to flee to Coimbatore, Tamil Nadu, India [2,3]. With the men at war, the females of the family stayed together throughout this time. Once her whole family reunited at the end of the war in 1945, Dr. Padmavati traveled to England to become a Fellow of the Royal College of Physicians, London, and the Royal College of Physicians of Edinburgh [4].

Lifelong learner

Since physicians, by nature, are wired to be lifelong learners, Dr. Padmavati's thirst for more knowledge led her to Sweden and then overseas to the United States. Here, she learned to perform surgeries on pediatric patients born with congenital heart disorders and worked under Dr. Helen Taussig. Dr. Padmavati then spent four years at Harvard Medical School training with Dr. Paul Dudley White, exposing her to new ideas and new technology, which she would later bring back to India [1,3]. During her time in the United States, Dr. Padmavati immersed herself in the world of modern cardiology and further developed her passion, working with fellow medical pioneers in the field.

The birth of cardiology in India

After learning abundant information from all her travels and further education worldwide, Dr. Padmavati returned to her roots and took her knowledge back to India. Upon her return, Dr. Padmavati was hand-chosen by the Union Health Minister to lecture at the Lady Hardinge Medical College (LHMC) in Delhi, India. After her time as a lecturer, Dr. Padmavati was promoted to professor and eventually became the head of the medicine department. During her time at the LHMC, Dr. Padmavati spearheaded the establishment of the first-ever Cardiac Catheterization Laboratory in North India [1-5]. She conducted research in the fields of epidemiology and public health. Tying in her research to her passion for the heart and love for cardiology, Dr. Padmavati focused on cardiovascular disease, hypotension, and coronary artery disease and their impacts on India [6].

**Figure 1:**
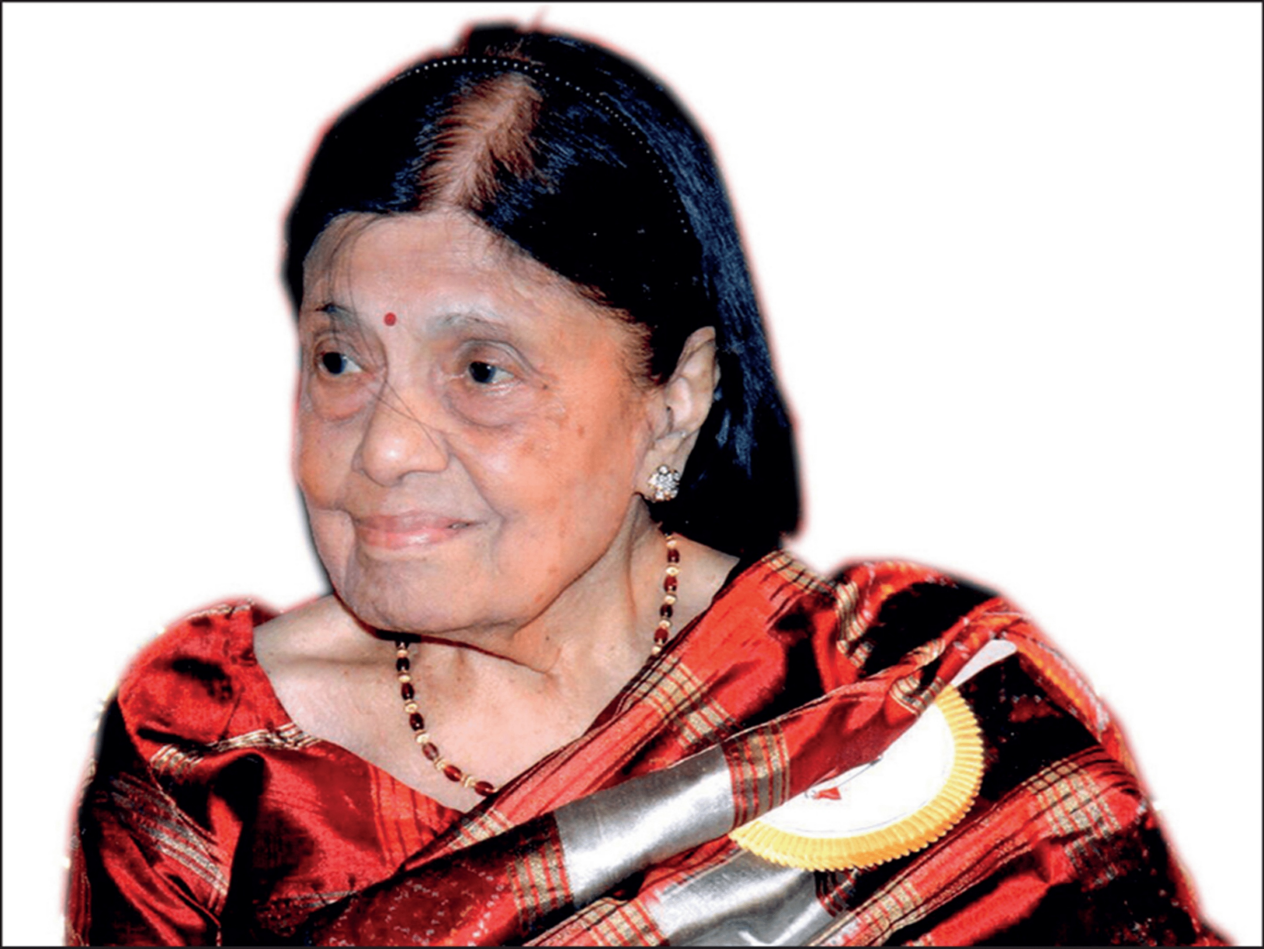
Dr. Sivaramakrishna Padmavati Iyer Reproduced with permission from Lancet via Elsevier RightsLink [11]

Dr. Padmavati (Figure 1) had many other prestigious leadership roles. She became the Director-Principal of Maulana Azad Medical College, where she built cardiology from the ground up. Adding to her long list of accolades, she became the president of the Cardiological Society of India and the National Academy of Medical Sciences [6]. She also founded the All India Heart Foundation, further promoting the field of cardiology in India and spreading awareness of heart disease [1-3]. In 1981, she founded the National Heart Institute, a part of the All India Heart Foundation, focused on research and training physicians in preventive cardiology [1,6].

Advocacy

Dr. Padmavati was a passionate advocate for a healthy lifestyle and preventative medicine, focusing on cardiovascular health. As a result of her expertise in cardiovascular disease, she provided extensive education to the Indian government. She even established the first cardiology department in a medical school [7]. She took many steps and paved the way for many educators, students, and, most importantly, her patients [6].

Later life

Dr. Padmavati never stopped her work in the field of medicine. Even at the age of 100, she continued with her rigorous routine working in academics, research, public health, and the clinic. She was the true embodiment of service, dedication, and passion. Staying committed to her love of public health, she worked tirelessly to promote heart health in India. Dr. Padmavati passed away from COVID-19 in August 2020, living to be 103 years old. She lived a long, fulfilled life, transforming the field of medicine in India. Although the end of her life marked the end of an era, her legacy will continue for generations to come, inspiring healthcare professionals and medical students worldwide. Dr. Padmavati's scholarly contributions include the publication of over 300 research articles in the field of preventive cardiovascular medicine. She is famously quoted as saying, "My mother lived to 105 and I followed her footsteps in adopting a healthy lifestyle. Remember, we are products of our environment" [6].

## Review

Dr. Padmavati received many honors and accolades during her long time in academia and the medical field. Beginning in 1967, after she had returned to India, she received the Padma Bhushan award [8]. This award is known as the third-highest civilian award in India and was given to her for her contributions to India. This award also came after she became Director-Principal at Maulana Azad Medical College [8]. In 1992, she was awarded the Padma Vibhushan award, which is the second-highest civilian award, and was given for advancing the cardiology departments in India. Due to Dr. Padmavati's monumental advancements in research, she was given a Fellowship from the European Society of Cardiology, along with the Emeritus Professor of Medicine and Cardiology, by the University of Delhi in 2007 [4]. In the following years, she would come to receive several other awards, which include the Harvard Medical International Award (2003), the Antonio Samia Oration of the Asia Pacific Society of Cardiology (2005), the Sivananda Eminent Citizen Award (2012), the Lifetime Award CSI (2012), the Lifetime Achievement Award of the National Academy of Medical Sciences (2013), and the Exceptional Service Award of the Golden Jubilee of the GBPH (2014) [4].

Along with prestigious awards, Dr. Padmavati also established the National Heart Institute in 1981 after establishing the All India Heart Foundation in 1962 [8-10]. This would then become the place for cardiac care and treatment for India's citizens. She single-handedly founded North India's first cardiac catheterization laboratory at the LHMC back in 1954 [4]. Dr. Padmavati's interest and passion for science continued all throughout her life, including her Fellowship in 2007 (at the age of 90) in the European Society of Cardiology [9,10].

## Conclusions

Dr. Padmavati left behind a legacy built upon the foundation of serving others and devoted her life to being a harbinger of positive change. As a pioneer in her own right, Dr. Padmavati helped to create several medical institutions in India throughout her lifetime and continued to look to the horizon for new endeavors to improve her profession. Credited for establishing cardiology in her home country and becoming the first female medical student at her medical college, she traveled to other nations to further expand her knowledge and ultimately returned to India to continue her passion for making lasting impacts. Even with her numerous accolades and living through times of war, Dr. Padmavati remained humble and family-oriented and was a trailblazer for more self-driven women in the field of medicine. Dr. Padmavati demonstrated incredible resilience, staying true to her values and overcoming numerous obstacles. She is a role model for aspiring physicians globally, and her legacy invokes inspiration for the next generation.
